# Serum periostin as a biomarker in eosinophilic granulomatosis with polyangiitis

**DOI:** 10.1371/journal.pone.0205768

**Published:** 2018-10-11

**Authors:** Rennie L. Rhee, Cecile T. J. Holweg, Kit Wong, David Cuthbertson, Simon Carette, Nader A. Khalidi, Curry L. Koening, Carol A. Langford, Carol A. McAlear, Paul A. Monach, Larry W. Moreland, Christian Pagnoux, Philip Seo, Ulrich Specks, Antoine G. Sreih, Steven R. Ytterberg, Peter A. Merkel, for the Vasculitis Clinical Research Consortium

**Affiliations:** 1 Division of Rheumatology, University of Pennsylvania, Philadelphia, Pennsylvania, United States of America; 2 Genentech Inc., South San Francisco, California, United States of America; 3 Department of Biostatistics, University of South Florida, Tampa, Florida, United States of America; 4 Division of Rheumatology, Mount Sinai Hospital, Toronto, Ontario, Canada; 5 Division of Rheumatology, St. Joseph’s Healthcare, McMaster University, Hamilton, Ontario, Canada; 6 Division of Rheumatology, University of Utah, Salt Lake City, Utah, United States of America; 7 Department of Rheumatology and Immunologic Disease, Cleveland Clinic, Cleveland, Ohio, United States of America; 8 Section of Rheumatology, Boston University School of Medicine, Boston, Massachusetts, United States of America; 9 Division of Rheumatology, University of Pittsburgh, Pittsburgh, Pennsylvania, United States of America; 10 Division of Rheumatology, Johns Hopkins University, Baltimore, Maryland, United States of America; 11 Division of Pulmonary and Critical Care Medicine, Mayo Clinic, Rochester, Minnesota, United States of America; 12 Division of Rheumatology, Mayo Clinic, Rochester, Minnesota, United States of America; 13 Division of Rheumatology and the Department of Biostatistics, Epidemiology, and Informatics, University of Pennsylvania, Philadelphia, Pennsylvania, United States of America; National and Kapodistrian University of Athens, GREECE

## Abstract

**Objective:**

Identification of a biomarker for disease activity in eosinophilic granulomatosis with polyangiitis (EGPA; Churg-Strauss) remains an unmet need. This study examined the value of serum periostin, a marker of type 2 inflammation, as a measure of disease activity in patients with EGPA.

**Methods:**

Participants enrolled in a multicenter, prospective cohort of patients with EGPA were included in this study if they had disease activity (defined as Birmingham Vasculitis Activity Score [BVAS] > 0) during follow-up. Serum levels of periostin were measured at flare visit as well as two pre- and two post-flare visits, if available. The outcome of disease activity was assessed either with BVAS or Physician Global Assessment (PGA). Mixed-effect models were used to examine the association between periostin levels and disease activity. Comparisons were made with a historical cohort of healthy individuals and patients with asthma.

**Results:**

In the 49 patients included in the study, the median periostin level was 60 ng/ml (IQR 50 to 73) in all visits and did not significantly change across visits. Multivariate analyses found no association between periostin level and presence or absence of flare according to the BVAS (adjusted OR 1.00 [95% CI 0.98 to 1.02], p = 0.98) but an increase in periostin level was significantly associated with greater disease severity during a flare according to the PGA (adjusted beta-coefficient 0.02 [95% CI 0.004 to 0.03], p = 0.01). Periostin levels in EGPA were significantly higher than previously studied healthy controls and patients with asthma.

**Conclusion:**

In EGPA serum periostin level is modestly associated with greater disease severity during a flare but does not discriminate active from inactive disease. Periostin levels in EGPA are higher than in other previously studied cohorts, including healthy populations and patients with asthma, and are relatively stable over time.

## Introduction

Eosinophilic granulomatosis with polyangiitis (EGPA, Churg-Strauss) is a multi-organ inflammatory disorder with common manifestations of asthma, rhinosinusitis, and peripheral blood eosinophilia[1]. Other potential areas of involvement include the lung parenchyma, nervous system, skin, gastrointestinal tract, and heart. While EGPA is considered a type of anti-neutrophil cytoplasmic antibody (ANCA)-associated vasculitis, only about 40% of patients have positive ANCA and vasculitic manifestations may not be present initially.

While therapeutic management has improved outcomes in EGPA, relapses remain a significant issue. In particular, distinguishing other disease activity from EGPA versus an isolated asthma exacerbation or infection is challenging and has important ramifications, including the escalation of potentially toxic immunosuppressive treatment. Similarly, differentiating active inflammation from permanent damage caused by prior disease is often difficult. Commonly used laboratory markers, such as blood eosinophil count, serum immunoglobulin E (IgE), erythrocyte sedimentation rate (ESR), or C-reactive protein (CRP), do not reliably discriminate active disease in EGPA from non-EGPA disease or damage, and are unpredictably influenced by glucocorticoids and other immunosuppressive therapies[2].

Serum periostin is a matricellular protein secreted by airway epithelial cells in response to stimulation by IL-13 and IL-4 and facilitates eosinophil-mediated type 2 inflammation and fibrosis, which are processes involved in EGPA[3–6]. The role of periostin in asthma and type 2 inflammatory responses has been increasingly recognized[7, 8]. Serum periostin has been associated with persistent eosinophilic airway inflammation in asthma[7] and higher frequency of asthma exacerbations[9]. It may also identify asthma patients who likely benefit most from therapies targeting type 2 inflammation like the IL-4Ra, IL-13 and IgE[10–13]. Periostin is also elevated in other eosinophilic syndromes, including eosinophilic otitis media and eosinophilic esophagitis[14, 15].

The objective of this study was to examine the role of serum periostin as a biomarker in patients with EGPA.

## Materials and methods

### Study population

Patients enrolled in the Vasculitis Clinical Research Consortium (VCRC) Longitudinal Study of EGPA were eligible for this study. The VCRC is comprised of over 15 international academic medical centers dedicated to conducting clinical and translational research in vasculitis. The Longitudinal Study of EGPA is an observational prospective cohort that collects clinical, laboratory, and blood samples of patients with EGPA at regular intervals (typically every 3 months) at 8 VCRC Centers in the United States and Canada. Patients are eligible to be enrolled in the cohort if they meet the American College of Rheumatology classification criteria for Churg-Strauss Syndrome (i.e., EGPA) and do not have any other inflammatory or autoimmune conditions[16]. This study was approved by the Institutional Review Board of the University of Pennsylvania and written informed consent was obtained from all participants.

For this study, disease activity was defined according to a validated disease activity index, the Birmingham Vasculitis Activity Score (BVAS)[17] with BVAS > 0 indicating active disease and BVAS = 0 indicating remission. Patients were included if they had a remission visit AND either active disease during a study visit (flare visit) or active disease between study visits. Patients with more than 1 distinct flare within 6 months were excluded (n = 2). Patients also had to have available serum samples corresponding to visits of interest in order to perform periostin testing.

### Exposure, outcomes, and co-variates

Serum levels of periostin were measured using the clinical trial version of the Elecsys^®^ Periostin assay (Roche Diagnostics, Penzberg Germany) which has been developed for clinical use and demonstrated to have high precision and reliability[18]. Absolute levels of periostin as well as changes in levels between 2 sequential visits (e.g., pre-flare to flare) were examined. The outcomes of interest were presence or absence of disease activity (i.e., flare vs remission) determined by BVAS (BVAS = 0 indicated remission and BVAS > 0 indicating active disease) and severity of flare according to Physician Global Assessment (PGA) score, which is the study physician’s assessment of severity of flare on a 10-point scale (score of 0 is remission and 10 is severe disease activity). Examples of a severe flare include any life- and/or organ-threatening manifestations such as cardiomyopathy, renal involvement, or neuropathy; mild flares include symptoms such as sinusitis, rash, or arthralgias. The BVAS and PGA are complementary but distinct: the BVAS is a count of disease manifestations during a flare and for this study was used to indicate presence or absence of active disease, regardless of disease severity. The PGA takes into account severity of disease activity. Both the BVAS and PGA scores are validated outcome measures which are highly reliable and correlated and no significant differences or outliers among the scores were found across 10 investigators in a validation study[17, 19]. During periods of remission, both BVAS and PGA are zero.

Co-variates and biospecimens were collected at time of visit according to protocol. Baseline (time of entry into cohort) demographics and disease characteristics (ANCA positivity ever and organ involvement any time since diagnosis) were obtained. At each visit, laboratory markers (including blood eosinophil count, serum immunoglobulin E, erythrocyte sedimentation rate, and C-reactive protein), disease activity and manifestations, and medications were recorded.

### Statistical analysis

Two-sample comparisons were performed using Wilcoxon rank sum test for continuous variables and chi-square test for categorical variables. Mixed-effect models examined the association between serum periostin levels (both absolute levels at each visit and changes in levels between visits) and disease activity, with the patient as the random effect to allow for assessment of changes within-patient. All models adjusted for ANCA type (ever positive or not), active asthma in the past 28 days, current prednisone use, and current use of other immunosuppressive therapies (cyclophosphamide, rituximab, azathioprine, methotrexate, or mycophenolate). Serum periostin levels for this cohort were compared using *Student’s* t-test to those of historical cohorts of healthy patients or patients with asthma based on previously published data using the same assay[9, 20]. A secondary analysis was performed between patients with EGPA who experienced an increase in serum periostin at time of flare visit from prior visit vs those who did not as well as between severe vs mild disease activity during flare.

## Results

There were 49 patients with EGPA with 186 visits who were included in this study (Table 1). All patients had a flare visit, 43 (88%) patients had at least 1 remission visit prior to the flare, and 37 (76%) patients had at least 1 remission visit after the flare. Forty-six of the 49 patients had active disease within the past 28 days while 3 patients had active disease since the prior visit but not within 28 days of the study visit. Similar to previously described cohorts, 29% of patients were myeloperoxidase (MPO)-ANCA positive, 51% had a history of a prior flare of EGPA, and the vast majority of patients had ear, nose, or throat (ENT) and lung involvement at some point in the disease course. At the time of the flare visit, more than half had ENT and/or lung involvement and the majority of patients were receiving some form of systemic immunosuppression.

**Table 1:** Characteristics of study patients with eosinophilic granulomatosis with polyangiitis.

	All N = 49	Low Periostin (< 50 ng/ml) N = 13	High Periostin (≥ 50 ng/ml) N = 36	P-value
***Baseline characteristics***				
Female	51%	46%	53%	0.68
White race	94%	100%	92%	0.56
MPO-ANCA positive (ever)	29%	38%	25%	0.36
Eosinophilia greater than 10% at diagnosis	92%	92%	92%	0.94
Elevated IgE level at diagnosis	45%	38%	47%	0.59
Prior history of flare of EGPA after achieving clinical remission	51%	69%	44%	0.13
***Clinical manifestations of EGPA at any time since diagnosis***				
Ear, nose, throat	94%	92%	94%	0.78
Lung	92%	100%	89%	0.21
Nervous system	61%	62%	61%	0.98
Skin	59%	69%	56%	0.39
Cardiac	22%	15%	25%	0.48
Kidney	14%	23%	11%	0.29
***Characteristics at flare visit***				
Age	53 (41 to 60)	53 (51 to 60)	53 (38 to 62)	0.70
BVAS	5 (2 to 7)	5 (4 to 7)	5 (2 to 8)	0.51
Serum periostin, ng/ml	60 (50 to 73)	45 (41 to 46)	69 (57 to 81)	< 0.01
Blood eosinophil count, 10^9^/L	0.47 (0.09 to 0.86)	0.29 (0.07 to 0.66)	0.53 (0.14 to 0.92)	0.35
Serum IgE level, mg/L	120 (22 to 277)	110 (4 to 250)	135 (22 to 374)	0.76
ESR, mm/hour	10 (4 to 22)	14 (8 to 27)	8 (4 to 21)	0.29
CRP, mg/L	2 (1 to 5)	3 (1 to 20)	2 (1 to 5)	0.25
Clinical manifestations of EGPA				
Ear, nose, throat	51%	46%	53%	0.68
Lung	65%	69%	64%	0.73
Nervous system	4%	8%	3%	0.44
Skin	8%	8%	8%	0.94
Cardiac	4%	8%	3%	0.44
Kidney	4%	8%	3%	0.44
***Medications at flare visit***				
Systemic glucocorticoids	74%	83%	71%	0.39
Inhaled glucocorticoids	62%	64%	61%	0.89
Cyclophosphamide	2%	0	3%	0.54
Rituximab	9%	8%	9%	0.98
Azathioprine	30%	50%	24%	0.09
Methotrexate	26%	33%	24%	0.48
Mycophenolate	7%	8%	6%	0.10

Values expressed as median (interquartile range [IQR]) or percentage

BVAS, Birmingham Vasculitis Activity Score. CRP, C-reactive protein. ESR, erythrocyte sedimentation rate. IgE, immunoglobulin E. MPO-ANCA, anti-myeloperioxidase antineutrophil cytoplasmic antibody.

**Fig 1:**
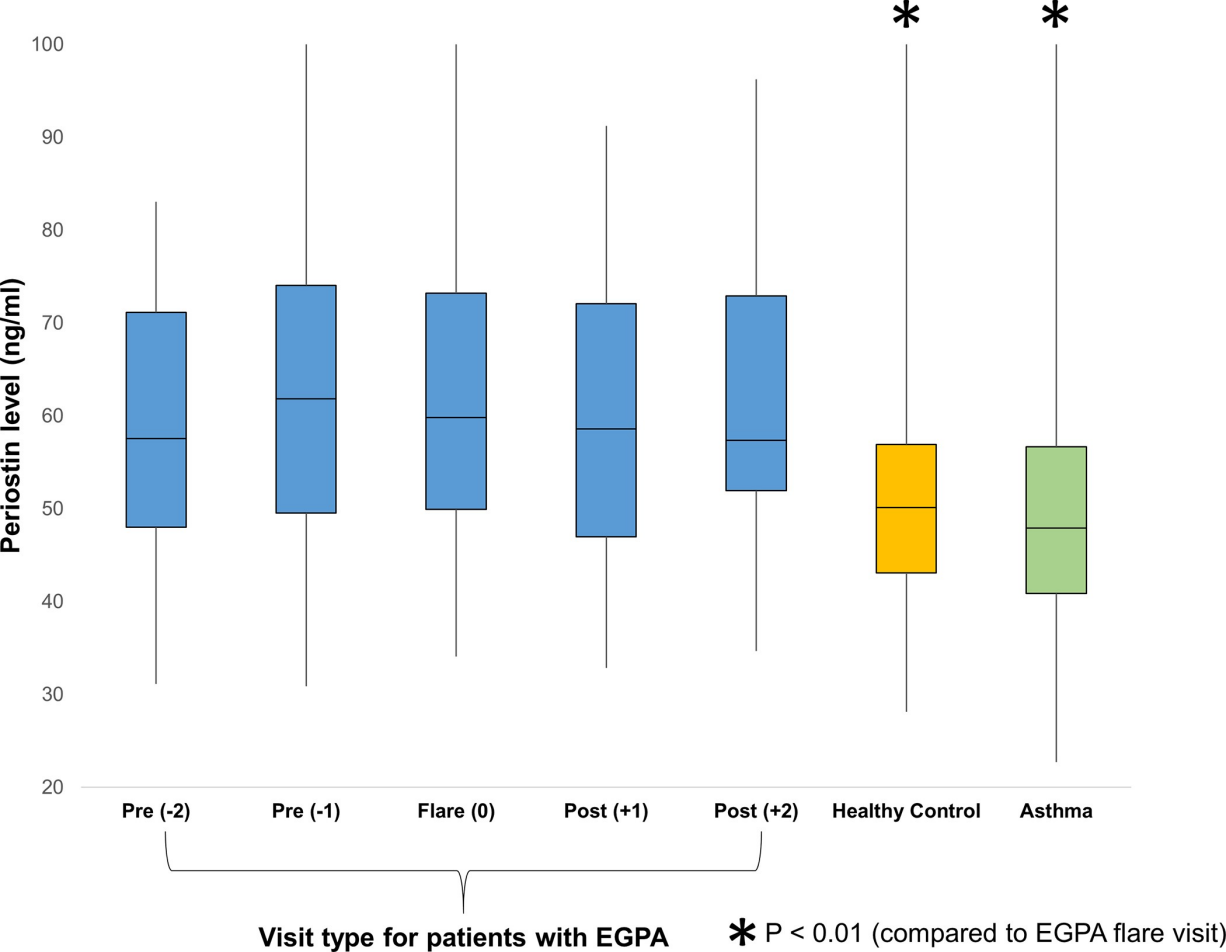
Median periostin level at all visits of patients with eosinophilic granulomatosis with polyangiitis (EGPA) compared to control groups. Visit types are arranged relative to the relapse visit (0). Periostin levels were stable across visits regardless of disease activity status. Levels for healthy control and asthma groups were obtained from previously-published data using same assay and were significantly lower compared to periostin levels of patients with EGPA who were relapsing (p < 0.01).

**Fig 2:**
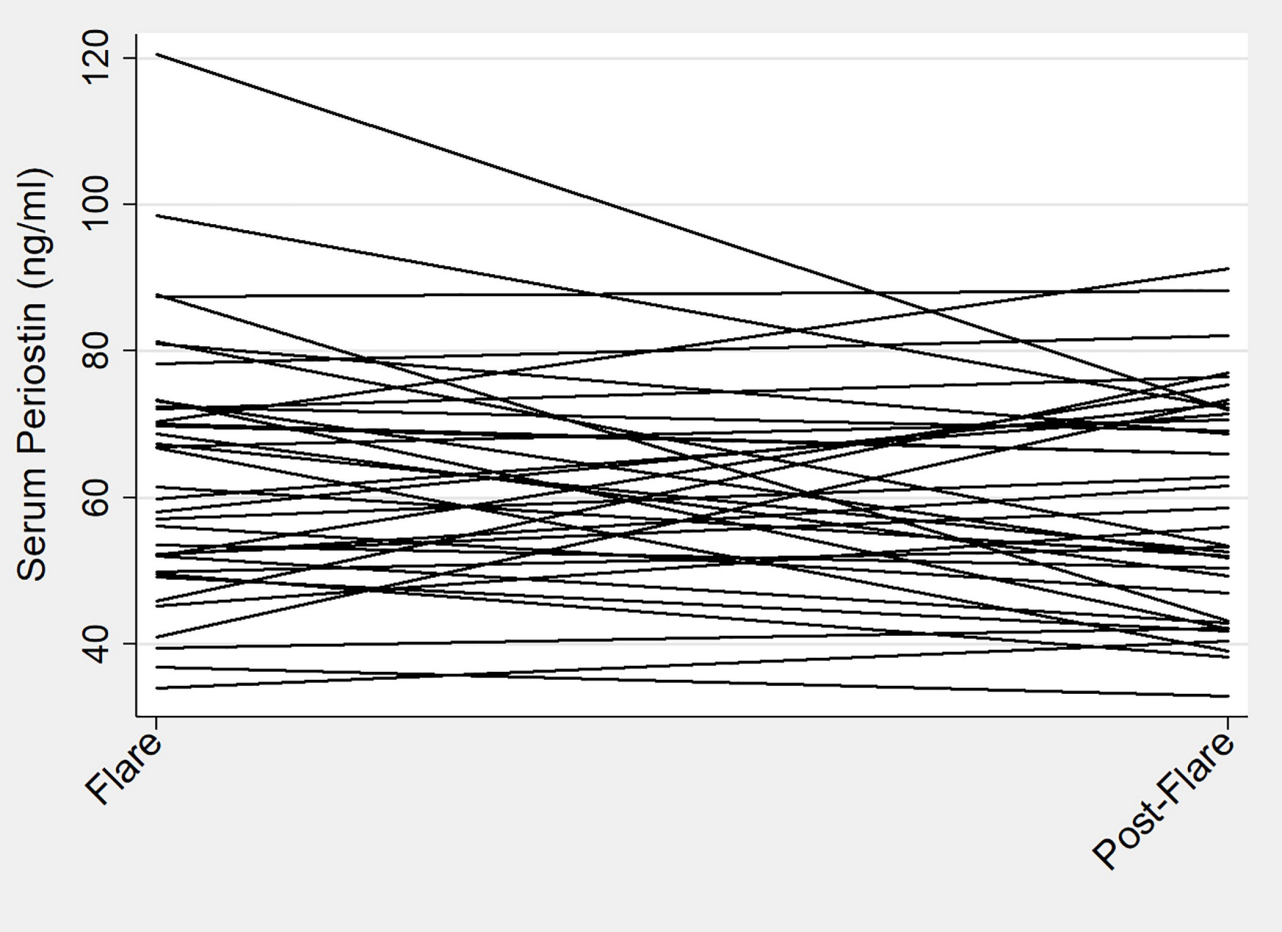
Individual participant periostin levels at flare and post-flare visit. This spaghetti plot depicts individual change in periostin level from flare to post-flare visit with a single line representing a single individual. Overall, no significant differences were observed in periostin level between flare and post-flare visit.

Median serum periostin level was 60 ng/ml (IQR 50 to 73) for all patients at all visits. Periostin levels remained fairly stable across visits regardless of disease activity status with median level of 60 ng/ml (IQR 50 to 73) for all flare visits and 59 ng/ml (IQR 50 to 72) for all remission visits (Fig 1). No significant changes were observed overall between the flare and post-flare visits (Fig 2). Patients with EGPA had significantly higher periostin levels when compared to previously published cohorts of healthy controls[20] (mean periostin 66 ng/ml [SD 25] vs 51 ng/ml [SD 12], p < 0.01) and patients with asthma[9] (mean periostin 66 ng/ml [SD 25] vs 51 ng/ml [SD 14], p < 0.01).

Multivariate analyses showed that periostin levels were not associated with a flare (yes vs no) visit after adjusting for baseline periostin level, ANCA type (ever positive or not), active asthma in the past 28 days, and use of immunosuppressive therapies (adjusted OR 1.00 [95% CI 0.98 to 1.02], p = 0.98)(Table 2). The median change in periostin level compared to the prior visit was 3 ng/ml (IQR -6 to 11) and change in periostin level between any 2 sequential visits was not associated with flare (yes vs no) visit (adjusted OR 0.99 [95% CI 0.97 to 1.01], p = 0.15). Furthermore, examination of BVAS as a continuous measure, instead of binary yes vs no for flare, also showed no association with periostin (beta-coefficient 0.004 [95% CI -0.024 to 0.032], p = 0.79).

**Table 2:** Relationship between serum periostin and disease activity in eosinophilic granulomatosis with polyangiitis.

Outcome	Exposure	Univariate analysis odds ratio or beta-coefficient (95% CI) p-value	Multivariate analysis odds ratio or beta-coefficient (95% CI) p-value
Flare visit (yes vs no)	Periostin level	OR 1.00 (0.99 to 1.02) p = 0.42	OR 1.00 (0.98 to 1.02) p = 0.98
	Change in periostin level	OR 0.99 (0.97 to 1.01) p = 0.30	OR 0.99 (0.97 to 1.01) p = 0.15
	Percent change in periostin level	OR 0.70 (0.17 to 2.91) p = 0.62	OR 0.43 (0.09 to 2.14) p = 0.30
Physician Global Assessment Score	Periostin level	beta 0.01 (0.002 to 0.02) p = 0.01	beta 0.02 (0.004 to 0.03) p = 0.01
	Change in periostin level	beta 0.01 (-0.002 to 0.02) p = 0.12	beta 0.01 (-0.005 to 0.02) p = 0.26
	Percent change in periostin level	beta 0.55 (-0.39 to 1.48) p = 0.25	beta 0.41 (-0.56 to 1.39) p = 0.40

Multivariate analyses adjusted for ANCA type (ever positive or not), active asthma in the past 28 days, current prednisone use, and current use of other immunosuppressive therapies; for all analyses except percent change in periostin level, baseline periostin also included in model.

CI, confidence interval. OR, odds ratio.

When the severity of disease activity was examined according to the PGA score at each study visit, there was an association between increased periostin level and a higher PGA score (adjusted beta-coefficient 0.02 [95% CI 0.004 to 0.03], p = 0.01) (Table 2). There was no association between change in periostin level and the PGA. When the analysis was restricted to only flare visits, periostin level remained significantly associated with PGA (adjusted beta-coefficient 0.03 [95% CI 0.007 to 0.05], p = 0.01). To determine if periostin level was different between mild vs severe flares, we examined the association between periostin level and severity of disease activity according to PGA score (PGA score < 5 is mild and PGA score ≥ 5 is severe). We found a non-significant increase in periostin level in patients with severe disease vs mild disease, although this association was not statistically significant (beta-coefficient 20 [95% CI -2 to 41], p = 0.07).

Among the subgroup of 20 patients who had an increase in serum periostin level between the pre-flare and flare visit, patients who experienced an increase in serum periostin had a higher median serum periostin level and blood eosinophil count at the time of the flare compared to those without a rise in periostin (Table 3). As expected, in univariate analysis blood eosinophil count is associated with serum periostin (for every 0.1 x 10^9^/L increase in blood eosinophil count, beta-coefficient 1.1 [95% CI 0.9 to 1.4], p < 0.01). At the flare visit patients with an increase in periostin level also had a higher PGA score and were less likely to be receiving azathioprine or inhaled glucocorticoids.

**Table 3:** Characteristics of patients with eosinophilic granulomatosis with polyangiitis during flare visits stratified by increase or no increase in serum periostin compared to prior visit*.

	Increase N = 20	No Increase N = 23	P-value
Periostin level	73 (56 to 87)	52 (46 to 64)	< 0.01
Change in periostin from prior visit	14 (5 to 21)	-11 (-27 to -3)	< 0.01
Blood eosinophil count, 10^9^/L	0.75 (0.19 to 1.39)	0.25 (0.07 to 0.53)	0.03
Serum IgE level, mg/L	37 (2 to 181)	181 (34 to 277)	0.16
ESR, mm/hr	6 (4 to 19)	10 (4 to 25)	0.42
CRP, mg/L	3 (0.5 to 5)	1 (0.7 to 3)	0.54
BVAS	4 (2 to 7)	5 (2 to 8)	0.75
Physician Global Assessment Score	3 (1 to 4)	1 (0 to 2)	< 0.01
***New or worse symptoms at flare (from BVAS)***			
Ear, nose, throat	65%	40%	0.10
Lung	65%	60%	0.72
Nervous system	4%	5%	0.92
Skin	4%	15%	0.23
Cardiac	4%	0%	0.35
Kidney	0%	5%	0.28
***Medications at time of flare***			
Any immunosuppressive	87%	100%	0.24
Systemic glucocorticoids	76%	89%	0.30
Cyclophosphamide	0%	5%	0.28
Rituximab	4%	11%	0.44
Azathioprine	4%	60%	< 0.01
Methotrexate	27%	25%	0.87
Mycophenolate	0%	10%	0.12
Inhaled glucocorticoid	52%	81%	0.07

Values expressed as median (IQR) or percentage

*6 patients did not have a preceding visit prior to the flare visit and were excluded from this analysis.

BVAS, Birmingham Vasculitis Activity Score. CRP, C-reactive protein. ESR, erythrocyte sedimentation rate. IgE, immunoglobulin E.

## Discussion

In EGPA, identifying active disease is often challenging especially when non-specific symptoms occur, such as worsening of asthma or rhinosinusitis. The development of predictive or diagnostic biomarkers would greatly improve the ability to determine who will or is currently relapsing. This study aimed to investigate the utility of serum periostin, which has been found to be elevated in other eosinophilic syndromes. This study found that while serum periostin does not discriminate active from inactive disease in EGPA, based on the standard disease activity index (BVAS), a higher periostin level is modestly associated with greater disease severity according to physician assessment. Furthermore, periostin levels are higher in EGPA compared to previously-studied cohorts of healthy populations and patients with asthma[9, 20].

Interestingly, two different measurements of disease activity yielded different results in this study. The BVAS is a useful tool which enumerates disease manifestations within the past 28 days while PGA is a summation measure based on the physician’s judgment. Therefore, it is possible, for example, that 2 patients with lung infiltrates will have the same BVAS but have different PGA scores if one patient has more severe symptoms and lung involvement than the other. The significant albeit modest association between periostin level and PGA but not BVAS suggests that periostin levels are sensitive to gradations in disease severity determined by the physician and no organ-specific involvement. In addition, the BVAS incorporates all manifestations within the past 28 days while the PGA score used in this study only evaluated disease activity on the day of the visit; thus, the use of immunosuppressive therapy (e.g., prednisone) since the onset of symptoms may have mitigated expected changes in periostin level.

While a statistically significant association was seen between PGA and periostin, the strength of the association was modest. This may be due in part to the mild severity of flares which may have contributed to less pronounced changes in periostin levels. As discussed previously, the large majority of patients in this cohort were receiving inhaled or oral immunosuppressive therapies which may also have affected results. Prior studies demonstrated that inhaled glucocorticoids affect serum periostin levels in asthma[5] and the current study found that patients on azathioprine were less likely to have an increase in periostin level at time of a flare. It is difficult, however, to know if the association between medications and periostin level are due to direct effects of immunosuppressives on periostin versus indirect effects mediated by better disease control (i.e., patients on immunosuppressives have less severe disease activity and therefore lower levels of periostin). Lastly, serum periostin levels can originate from sources other than the airway, further confounding results.

Notably, while the periostin levels were fairly stable across visits, they were overall much higher than previously-described cohorts, including patients with asthma, despite the concurrent use of immunosuppressive therapy. The use of periostin in distinguishing EGPA from other eosinophilic syndromes, including isolated asthma, deserves further exploration.

There were several limitations to this study. Asthma and sinusitis, which in isolation are not considered to be features of active disease in EGPA, were poorly characterized and may have been important confounders in the analysis. Disease activity measurements relied in part on investigator judgment and may have been less sensitive to subclinical or low levels of disease activity. Lastly, as discussed above, the majority of patients were receiving immunosuppressive therapy during the flare visit which may further have dampened the performance of the biomarker. Dosage of immunosuppressive therapies was not available to further understand effects of medications on periostin level. However, this study closely resembled a real-world setting and, therefore, more accurately characterizes the effectiveness of periostin as a biomarker.

Despite these limitations, the major strength of this study was its use of a fairly large, prospective longitudinal cohort of patients with this rare disease and with assessment and data collection performed by experts in EGPA. Standardized forms were used to collect data such as disease activity, medications, and laboratory values. Serum periostin in all samples was measured with a validated assay that was developed for clinical use.

In conclusion, serum periostin level is modestly associated with severity of disease activity during flare according to the PGA score but does not discriminate active from inactive disease. Periostin is significantly higher in EGPA compared to healthy populations and patients with asthma. Further investigation in the utility of periostin as a biomarker in severe EGPA and differentiation of EGPA from other eosinophilic syndromes is warranted, perhaps in combination with other biomarkers or clinical variables.

## Supporting information

S1 Dataset De-identified dataset. (XLS)
